# Expression analysis of NF-ƙB-related long non-coding RNAs in bipolar disorder

**DOI:** 10.1038/s41598-022-25670-9

**Published:** 2022-12-03

**Authors:** Sara Ahmadi Teshnizi, Pariya Shahani, Mohammad Taheri, Bashdar Mahmud Hussen, Solat Eslami, Zahra Sadeghzadeh, Soudeh Ghafouri-Fard, Arezou Sayad

**Affiliations:** 1 Department of Medical Genetics, School of Medicine, Shahid Beheshti University of Medical Sciences, Tehran, Iran; 2 Department of Cellular and Molecular Biology, Faculty of Advanced Science and Technology, Tehran Medical Sciences, Islamic Azad University, Tehran, Iran; 3 Men’s Health and Reproductive Health Research Center, Shahid Beheshti University of Medical Sciences, Tehran, Iran; 4 Institute of Human Genetics, Jena University Hospital, Jena, Germany; 5 Department of Biomedical Sciences, Cihan University-Erbil, Kurdistan Region, Erbil, Iraq; 6 Department of Pharmacognosy, College of Pharmacy, Hawler Medical University, Kurdistan Region, Erbil, Iraq; 7 Department of Medical Biotechnology, School of Medicine, Alborz University of Medical Sciences, Karaj, Iran; 8 Dietary Supplements and Probiotic Research Center, Alborz University of Medical Sciences, Karaj, Iran

**Keywords:** Genetics, Molecular biology, Neuroscience

## Abstract

Bipolar disorder (BD) is a mental disorder that leads to abnormal swings in mood, energy, activity level, attention, and the capability to accomplish daily tasks. Several long non-coding RNAs (lncRNAs) are dysregulated in BD patients. We have compared expression levels of five NF-κB-associated lncRNAs, namely *ANRIL*, *CEBPA-DT*, *H19*, *NKILA* and *HNF1A-AS1* in blood samples of BD patients compared with controls. While *ANRIL*, *CEBPA-DT* and *HNF1-AS1* were significantly under-expressed in BD patients compared with controls, *NKILA* levels were higher in patients versus controls. Among differentially expressed genes, *HFN1A-AS1* exhibited the best diagnostic parameters in the separation of patients from controls (AUC ± SD = 0.86 ± 0.03, sensitivity = 0.82, specificity = 0.82, *P* value < 0.0001). AUC values for *NKILA*, *ANRIL* and *CEBPA-DT* were 0.71, 0.68 and 0.65, respectively. In accordance with the previously reported participation of NF-ƙB in the pathophysiology of BD, the current study provides evidence for dysregulation of NF-κB-associated lncRNAs in BD.

## Introduction

Bipolar disorder (BD) is a mental disorder that leads to abnormal swings in mood, energy, activity level, attention, and the capability to accomplish daily tasks^1^. BD can be classified into two main categories, namely BD type I and type II with severe and persistent mood elevation in the former type but less severe mood elevation in the latter type^1^. BD, particular type I has been shown to have a strong genetic background^2^. Based on the results of family studies, it seems that a small number of genes with modest effects establish genetic background of BD^2^. In addition, a number of linked chromosomal regions as well as candidate genes have been identified^2^. Moreover, expression assays have shown dysregulation of several genes in the circulation of BD patients or in the postmortem brain samples^3–5^. Ubiquitin cycle, synaptic function^3^, apoptosis regulators^5^ and vitamin D related genes^6^ are among dysregulated genes in BD.

Nuclear factor kappa B (NF-κB) is an important transcription factor which regulates inflammatory signals. Expression of this transcription factor has been dysregulated in BD. Elevation in the spontaneous levels of NF-κB have been reported in several types of immune cells in adolescents with BD. Moreover, BD patients have exhibited greater upsurges in the NF-κB levels in monocytes after induction with TNF-α. Most notably, the latter observation has been associated with the contemporary severity of depressive symptoms^7^.

Several non-coding RNAs have exhibited functional association with NF-κB^8^. Association of number of NF-κB-interacting long non-coding RNAs (lncRNAs) with human disorders has been more investigated. LncRNAs represent a group of transcripts with sizes more than 200 nucleotides that contribute in the regulation of gene regulation. These transcripts participate in the pathogenesis of several human disorders through changing expression of genes and influencing activity of signaling pathways^9,10^. Among these lncRNAs are ANRIL, CEBPA-DT, H19, NKILA and HNF1A-AS1 whose participation in the pathogenesis of Parkinson's disease^11^ and autism spectrum disorder^12^ has been assessed. In the current study, we measured expression levels of these lncRNAs in the blood samples of BD patients compared with controls to appraise their possible contribution in this disorder. We hypothesized that expression of these lncRNAs has been changed in the peripheral blood of BD patients due to the abnormalities in gene regulation pathways. Since they are associated with NF-κB signaling, it is possible that they contribute to the pathoetiology of BD. For instance, ANRIL as a constituent of NF-κB pathway can regulate inflammatory response^13^. H19 has been shown to promote atherosclerosis through regulation of MAPK and NF-κB pathways^14^. NKILA has also been identified as an inhibitor of NF-κB, since it inhibits phosphorylation of IκBα and suppresses nuclear translocation of p65^15^. CEBPA-DT is another lncRNA that is transcribed from up-stream of the CEBPA gene regulating its expression *in cis*^16^. Notably, expression of CEBPA has also been shown to be regulated by NF-κB p50 ^17^. Besides, CEBPA contributes to the relocation of histone deacetylases from NF-κB p50 homodimers and induction of expression of NF-κB target genes^18^. Finally, HNF1A-AS1 is an NF-κB-regulated lncRNA that enhances the phosphatase activity of SHP-1^19^. Notably, this phosphatase has a role in the pathogenesis of immune-related disorders^20^.

## Material and methods

### Subjects

The Ethical Committee of Shahid Beheshti University of Medical Sciences has confirmed the study protocol (IR.SBMU.MSP.REC.1400.620). All enrolled cases and controls signed the written informed consent. Blood samples of 50 type I BD patients and 50 normal subjects were collected from Imam Hussein hospital during 2016–2019. Cases were recruited consecutively from Imam Hossein hospital during the study period and were examined in this hospital. BD cases were diagnosed according to the Diagnostic and Statistical Manual of Mental Disorders-5^21^. Moreover, depressive and mania symptoms and the presence of euthymia were assessed using the Hamilton Depression Rating Scale (HAM-D)^22^ and Young Mania Rating Scale (YMRS)^23^. Patients were also assessed using a semi-structured interview which was based on asking questions within a prearranged thematic structure by skilled specialists who were trained through passing relevant courses. Clinical data including disease duration/ onset and drug history were collected. All recruited patients were not responsive to first-line mood stabilizers and took standard dose of Carbamazepine (200 mg, 2 times a day). In fact, most of patients who were not responsive to the first-line mood stabilizers in the mentioned clinic were under treatment with carbamazepine. So, in order to have a homogenized cohort of patients, we just included these patients. With the purpose of decreasing heterogeneity of the patients’ cohort, cases who took other drugs were excluded. History of head trauma, encephalitis or other mental illnesses and systemic disorders were regarded as exclusion criteria. Control subjects were assessed by a specialist to rule out the presence of signs or symptoms related with psychiatric disorders.

### Sample collection and RNA extraction

Five ml of peripheral blood was collected from all cases and controls. Total RNA was extracted from blood samples using RNX kit (EX6101, Cinnagen, Tehran, Iran). Extracted RNA was assessed using gel electrophoresis and spectrophotometer.

### cDNA production and real-time PCR assay

cDNA was made using High-Capacity cDNA Reverse Transcription Kit (Applied Biosystems), based on the Company's comments. Expression levels of *ANRIL*, *CEBPA-DT*, *H19*, *NKILA* and *HNF1A-AS1* were enumerated in comparison with *beta 2 microglubolin* (*B2M*) as an internal control using self-designed primers which were similar to our previous study^24^. qRT-PCR was implemented in the ABI 7500 sequence detection system (Applied Biosystem, Foster City, CA, USA) using BIOFACT™ 2X Real-Time PCR Master Mix. All experiments were conducted in duplicate. Table 1 shows the primer sequences. Primers were synthetized by Macrogen Company (Seoul, Korea). Transcripts were enumerated using the comparative –delta Ct method. The primers were designed to amplify 14 isoforms of *ANRIL* and 3 isoforms of *H19*. For other lncRNAs, only one isoform was amplified by designed primers.

**Table 1:** Information about primers and the amplified transcripts.

Gene	Primer sequence		Product size (bp)	Amplified isoforms
HNF1A-AS1	Forward	CCAGCCTGACCTCTCCATTCC	158	NR_024345.1 (NCBI). ENST00000539163.1 (Ensembl)
	Reverse	GCCGAACTGACATCACTGAACAC		
NKILA	Forward	AACCAAACCTACCCACAACG	108	NR_131157.1. ENST00000614771.2
	Reverse	ACCACTAAGTCAATCCCAGGTG		
ANRIL	Forward	TGCTCTATCCGCCAATCAGG	108	NR_003529.3, transcript variant 1. ENST00000428597.6 NR_047532.1, transcript variant 2 . ENST00000580576.6 NR_047543.1, transcript variant 3. ENST00000582072.5 NR_047534.1, transcript variant 4. ENST00000585267.5 NR_047535.1, transcript variant 5. ENST00000581051.5 NR_047536.1, transcript variant 6 . ENST00000577551.5 NR_047537.1, transcript variant 7. ENST00000584637.5 NR_047538.1, transcript variant 8. ENST00000584020.5 NR_047539.1, transcript variant 9. ENST00000584351.5 NR_047540.1, transcript variant 10. ENST00000580467.5 NR_047541.1, transcript variant 11. ENST00000582301.5 NR_047542.1, transcript variant 12. ENST00000455933.7 NR_047533.1, transcript variant 13. ENST00000583719.5 NR_120536.1, transcript variant 14. ENST00000584816.5
	Reverse	GCGTGCAGCGGTTTAGTTT		
CEBPA-DT (ADINR)	Forward	TGGATGTGCTGTGATGAAGAGAAG	91	NR_026887.2. ENST00000592982.2
	Reverse	CCATAACACCTCCGCAGACAAATC		
H19	Forward	TGCTGCACTTTACAACCACTG	101	NR_002196.2, transcript variant 1. ENST00000414790.8 NR_131223.1, transcript variant 2. ENST00000414790.8 NR_131224.1, transcript variant 3. ENST00000428066.7
	Reverse	ATGGTGTCTTTGATGTTGGGC		
B2M	Forward	AGATGAGTATGCCTGCCGTG	105	NM_004048.4. ENST00000617605.4
	Reverse	GCGGCATCTTCAAACCTCCA		

### Statistical methods

GraphPad Prism version 9.0 (La Jolla, CA, USA) was used for statistical analysis. We compared expression levels of five lncRNA genes, namely *ANRIL*, *CEBPA-DT*, *H19*, *NKILA* and *HNF1A-AS1* in blood samples obtained from BD patients and controls. Normal/Gaussian distribution of the values was evaluated using the Shapiro–wilk test. As data was not normally distributed, we used the non-parametric Mann–Whitney U test to detect differentially expressed transcripts between cases and controls. Two-way ANOVA test and Tukey post hoc test (as a complementary test for ANOVA test) were used for assessment of the effect of disease and gender on expression of genes and their interactions.

Correlations between gene expression levels of lncRNAs were measured with Spearman’s rank correlation coefficient as data was not normally distributed. In addition, correlations between gene expression levels and age, disease duration, sex and age at onset were measured using the Spearman’s rank correlation coefficient.

Receiver operating characteristic (ROC) curves were illustrated to judge the diagnostic power of expression levels of differentially expressed lncRNAs. *P* value < 0.05 was considered as significant.

### Ethical approval and consent to participant

All procedures performed in studies involving human participants were in accordance with the ethical standards of the institutional and/or national research committee and with the 1964 Helsinki declaration and its later amendments or comparable ethical standards. Informed consent forms were obtained from all study participants. Informed consent forms were obtained from all study participants. The study protocol was approved by the ethical committee of Shahid Beheshti University of Medical Sciences. All methods were performed in accordance with the relevant guidelines and regulations.

## Results

### General information about cases and controls

Table 2 shows age, sex ratio and other available information about enrolled persons. There was no significant difference in age between cases and controls (*P* value = 0.11).

**Table 2:** General data of cases and controls.

Study group	Parameters	Values	
Patients	Sex (number)	Male	35
		Female	15
	Age (Years, mean ± SD)	Male	38.48 ± 9.33
		Female	31.87 ± 7.73
	Duration (Years, mean ± SD)	Male	4.25 ± 2.77
		Female	2.86 ± 2.16
	Age of onset (Years, mean ± SD)	Male	34.2 ± 8.21
		Female	29 ± 6.49
Controls	Sex (number)	Male	35
		Female	15
	Age (Years, mean ± SD)	Male	33.25 ± 8.84
		Female	34.46 ± 8.21

### Expression assays

**Figure 1:**
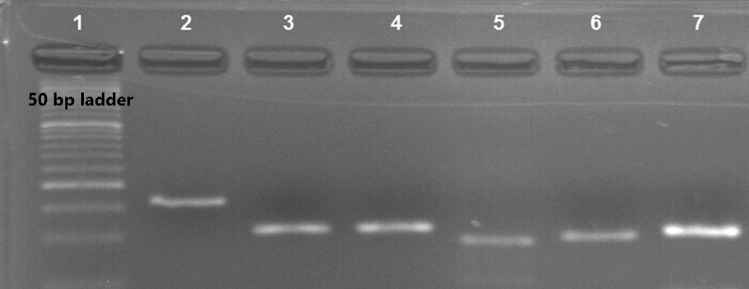
RT-PCR products were electrophoresed on 2% agarose gel. Lanes 1–7 are 50 bp ladder, *HNF1A-AS1*, *NKILA*, *ANRIL*, *CEBPA-DT* (*ADINR*), *H19* and *B2M*, respectively.

Since we hypothesized that expression of NF-ƙB-related lncRNAs would be different among BD patients and controls, we assessed their expression in the peripheral blood of both study groups using real time PCR method. Figure 1 shows the corresponding band to the assessed lncRNAs as well as the housekeeping gene after RT-PCR.

**Figure 2:**
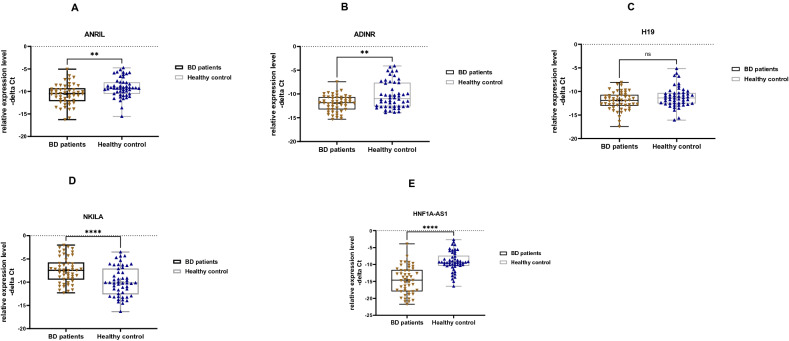
Relative expression levels of five lncRNAs in bipolar disorder (BD) patients (total) and healthy controls (total) as described by –delta Ct values (**A**–**E**).—delta Ct values were plotted as box and whisker plots showing median, mean, interquartile range, and minimum and maximum values. Mann–Whitney U test was used to detect differentially expressed genes between cases and controls (**P value < 0.01 and ****P value < 0.0001).

We detected significant difference in expression of *ANRIL*, *CEBPA-DT* (*ADINR*), *NKILA* and *HNF1A-AS1* between BD patients and controls (Fig. 2).

Group (disease) factor had significant effect on expression levels of *ANRIL*, *CEBPA-DT*, *HNF1-AS1* and *NKILA*. However, neither gender factor nor interaction of gender and group has significant effects on expression levels of studied gene; therefore, we did not perform post hoc tests for multiple comparisons (Table 3).

**Table 3:** Graphpad prism output from showing the effects of Group and Gender (Tests of Between-Subjects Effects) on levels of lncRNAs in cases compared to healthy controls.

Source of Variation	Group effect			Gender effect			Interactions		
	SS^1^	F^2^	*P* value	SS	F	*P* value	SS	F	*P* value
ANRIL	57.09	11.34	0.0011*	1.1e-005	2.3e-006	0.99	3.48	0.69	0.4
CEBPA-DT	89.66	16.54	< 0.0001*	0.77	0.14	0.7	16.01	2.95	0.08
H19	12.34	2.9	0.09	4.16	0.98	0.32	0.21	0.05	0.82
NKILA	106.8	12.25	0.0007*	12.08	1.38	0.24	6.27	0.72	0.39
HNF1A-AS1	590.5	46.48	< 0.0001*	1.95	0.15	0.69	3.47	0.27	0.6

^1^Sum of Squares.

^2^F of Variance.

Significant values are in bold.

While *ANRIL*, *CEBPA-DT* and *HNF1A-AS1* were significantly under-expressed in BD patients compared with controls, *NKILA* levels were higher in patients versus controls.

In order to assess possible relation between expressions of mentioned lncRNAs, we performed correlation analysis in each study subgroup. We detected significant pairwise correlation between all lncRNA pairs among BD patients and healthy controls (Table 4).

**Table 4:** Spearman’s correlation between RNA expression levels among BD patients (*N* = 50) and healthy controls (*N* = 50).

	CEBPA-DT		H19		NKILA		HNF1A-AS1	
	Cases	Controls	Cases	Controls	Cases	Controls	Cases	Controls
ANRIL	0.3*	0.7**	0.37*	0.42*	0.25	0.59**	0.3*	0.5**
CEBPA-DT			0.57**	0.63**	0.59**	0.77*	0.3*	0.65**
H19					0.55**	0.55**	0.53**	0.53**
NKILA							0.46**	0.73**

*p < 0.05.

**p < 0.001.

**Figure 3:**
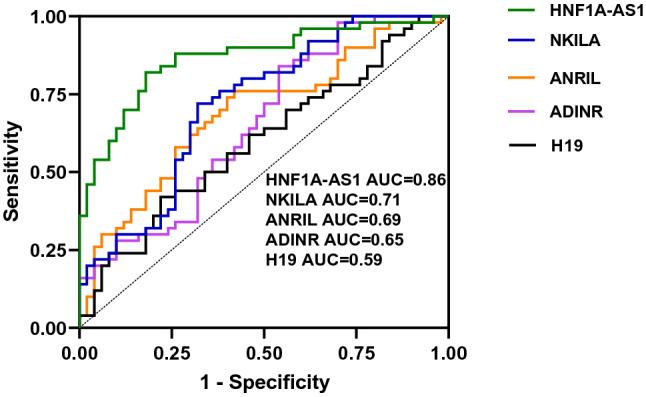
ROC curves of transcript levels of five lncRNAs in patients with bipolar disorder.

In order to assess the possibility of using expression levels of mentioned lncRNAs as diagnostic markers in BD, we assessed their diagnostic power using ROC curve analysis. Among differentially expressed genes, *HFN1A-AS1* exhibited the best diagnostic parameters in separation of patients from controls (AUC ± SD = 0.86 ± 0.03, sensitivity = 0.82, specificity = 0.82, *P* value < 0.0001). AUC values for *NKILA*, *ANRIL* and *CEBPA-DT* were 0.71, 0.68 and 0.65, respectively (Fig. 3 and Table 5).

**Table 5:** ROC curve analysis for 4 differentially expressed lncRNAs in patients with BD disorder.

HFN1A-AS1				NKILA				ANRIL				CEBPA-DT			
AUC ± SD	Sensitivity	Specificity	*P* Value	AUC ± SD	Sensitivity	Specificity	*P* Value	AUC ± SD	Sensitivity	Specificity	*P* Value	AUC ± SD	Sensitivity	Specificity	*P* Value
0.86 ± 0.03	0.82	0.82	< 0.0001	0.71 ± 0.05	0.72	0.68	0.0002	0.68 ± 0.05	0.74	0.6	0.0012	0.65 ± 0.05	0.84	0.46	0.008

Finally, we checked whether expression of mentioned lncRNAs is different between male and female subgroups or whether their expression is correlated with demographic/clinical parameter. In spite of significant difference in expression of four lncRNAs between cases and controls, expression levels of none of lncRNAs were correlated with clinical/demographic parameters (Table 6).

**Table 6:** The results of Spearman’s rank correlation between expression of five lncRNAs, and clinical/demographic data, including age, disease duration, sex and age at onset.

	Age	Sex	Disease duration	Age at onset
ANRIL	− 0.112	0.11	− 0.055	− 0.125
CEBPA-DT	− 0.046	0.27	− 0.028	− 0.055
H19	− 0.128	0.08	0.126	− 0.184
NKILA	− 0.045	0.2	− 0.03	− 0.027
HNF1A-AS1	0.03	− 0.1	0.189	− 0.022
Age		0.34*	0.49**	0.97**
Sex			0.27	0.3*
Disease duration				0.34*

*Significance level of *p* < 0.05.

**Significance level of *p* < 0.001.

Disease duration was classified into 3 ranges (1–2, 3–4 and more than 4 years).

Significant values are in bold.

## Discussion

NF-κB pathway has an indispensable part in the development of innate and adaptive immunity^25^, which are dysregulated in BD^26^. The impact of NF-κB signals in inflammation, neuroprotection, and apoptosis are particularly obvious in the nervous system^27^. NF-κB can also modulate neuronal excitability and susceptibility to excitotoxicity^28^. In addition, pro-inflammatory cytokines which are associated with NF-κB can influence the process of neuroplasticity^28,29^. An early study in the postmortem tissue samples of BD patients has demonstrated elevation of NF-κB2 in BD samples. Based on the reported impact of viruses and cytokines on expression of NF-κB2, authors have suggested that up-regulation of NF-κB2 is in line with the contribution of possible environmental factors in the pathogenesis of BD^30^. Subsequently, Rao et al. have demonstrated up-regulation of NF-κB in the BD brain samples consistent with the elevation of various inflammatory cytokines, induction of apoptosis, brain atrophy and cognitive deficits in these patients^31^.

We have assessed expression levels of NF-κB-related lncRNAs in BD patients and healthy subjects. While *ANRIL*, *CEBPA-DT* and *HNF1-AS1* were significantly under-expressed in BD patients compared with controls, *NKILA* levels were higher in patients versus controls. *ANRIL* has been revealed to regulate inflammatory response as a constituent of NF-κB pathway^13^. We have recently reported association between rs1333048 variants of *ANRIL* and risk of BD I in an Iranian cohort of patients. Moreover, rs1333045 and rs1333048 variants of *ANRIL* have been associated with risk of BD II. Moreover, T A haplotype block (rs1333045 and rs1333048, respectively) has been found to decrease risk of BD I and II, while C C haplotype decreases risk of BD II^32^. Thus, the present study shows further evidence for participation of *ANRIL* in the pathoetiology of BD.

We have also previously reported up-regulation of *HNF1A-AS1* in patients with schizophrenia compared with controls^24^. Thus, this lncRNA might influence pathogenesis BD and schizophrenia in different directions.

Among differentially expressed genes, *HFN1A-AS1* exhibited the best diagnostic parameters in separation of patients from controls, potentiating this lncRNA as a possible biomarker for BD.

We also reported correlations between expression levels of these lncRNAs in both patient and control groups, providing evidence for our hypothesis regarding association between these lncRNAs and NF-κB. However, expression of none of lncRNAs was associated with clinical and demographic data of BD patients. Our study has limitations regarding sample size, lack of drug-naïve patients, lack of access to HAM-D and YMRS scores, unavailability of history of drugs other than antipsychotic drugs, lack of functional studies and verification of the obtained results with other techniques. Expression of genes might be affected by administration of carbamazepine. The small sample size might affect the significance of obtained data. Finally, functional studies are needed to find the mechanistical points about contribution of mentioned genes to the pathogenesis of BD.

Taken together, this study provides evidence for participation of NF-κB-associated lncRNAs in BD and warrants additional functional studies. Future studies involving patients in different phases (i.e., mania, depression) should be performed in order to evaluate whether the lncRNA changes are independent of the disease phase. Moreover, based on the literature-based approach that was used for selection of mentioned lncRNAs, we just can conclude functional interactions between these lncRNAs and NF-κB signaling, possibly downstream or upstream of this pathway. Further assays should find their exact role on the activity of NF-κB signaling.

## Supplementary Information

Supplementary Information.

## Data Availability

All data generated or analysed during this study are included in this published article [and its supplementary information files].
